# Promoting active aging through assistive product design innovation: a preference-based integrated design framework

**DOI:** 10.3389/fpubh.2023.1203830

**Published:** 2023-06-19

**Authors:** Baoyi Zhang, Minyuan Ma, Zongsheng Wang

**Affiliations:** ^1^School of Design Art, Xiamen University of Technology, Xiamen, China; ^2^Department of Industrial Design, National Cheng Kung University, Tainan, Taiwan

**Keywords:** active aging, assistive products, preference-based design, Miryoku engineering, evaluation grid method (EGM), universal design (UD), TRIZ

## Abstract

**Background:**

With the accelerating trend of global aging, over one billion people need to use one or more types of assistive products. However, the high abandonment rate of current assistive products is affecting the quality of life of the older adults, posing challenges to public health. Accurately capturing the preference factors of the older adults in the design process is an important way to improve the acceptance of assistive products. In addition, a systematic approach is needed to translate these preference factors into innovative product solutions. These two issues are less addressed in existing research.

**Methods:**

First, the evaluation grid method was used to conduct in-depth interviews with users and extract the structure of preference factors for assistive products. Quantification theory type I was used to calculate the weight of each factor. Secondly, universal design principles, TRIZ theory's contradiction analysis techniques, and invention principles were used to translate the preference factors into design guidelines. Then, finite structure method (FSM), morphological chart, and CAD techniques were used to visualize the design guidelines as alternatives. Finally, Analytic Hierarchy Process (AHP) was used to evaluate and rank the alternatives.

**Results:**

A Preference-based Assistive Product Design Model (PAPDM) was proposed. The model includes three stages: definition, ideation, and evaluation. A case study on walking aid demonstrated the execution of PAPDM. The results show that 28 preference factors influence the four psychological needs of the older adults: sense of security, sense of independence, self-esteem, and sense of participation. These psychological needs were reflected in the shape, color, material, universality, user-friendly, reliability, and smart functions of assistive products. The preference factors were transformed into five design guidelines, and three alternatives were generated. Finally, the evaluation concludes that solution C was the optimal solution.

**Conclusion:**

The PAPDM framework provides designers with a transparent, progressive approach to designing assistive products that meet unique needs and preferences of older adults. This enhances objectivity and scientific rigor in assistive product development, avoiding blind design and production. By considering the perspective of older adults from the outset, we can avoid high abandonment rates of assistive products and contribute to promoting active aging.

## 1. Introduction

The pace of population aging is much faster than in the past. according to the WHO, Between 2015 and 2050, the proportion of the world's population over 60 years will nearly double from 12 to 22% (1). However, as people's life expectancy increases, poor health conditions become more common (2). This issue poses a major public health challenge (2–8).

Promoting active aging has become a timely policy response for these countries in response to the challenges posed by accelerated population aging (9). Extensive research has shown that assistive technology (AT) can promote important dimensions of active aging (e.g., physical health, mental health, social participation, and lifelong learning) (6–8, 10–12). It is a fundamental part of broader, integrated health and social system solutions for supporting older adults (13).

Assistive products (Aps) are an essential component of the implementation of assistive technology policies (14) and are also considered to be an important contribution to public health. The use of APs is a common strategy for community elders to maintain their independence and cope with daily activities (15). over one billion people need one or more assistive products. The majority of these are older people and people with disabilities (16). And that number is expected to increase to more than 2 billion by 2050. However, due to cost, availability and financial issues, only about 10% of those in need have access to these products. This leads to impairment of the ability to perform activities of daily living (ADLs) and reduced life satisfaction (LS) (2) in Older Adults (OA). Effective tools and outcomes have been developed to help increase the accessibility of assistive products for older adults, including the Priority Assistive Product List (APL) developed by WHO (16), Assistive Product Explorer (ASPREX) from Global Collaboration On Assistive Technology (GATE) (17), International standards for Assistive products (ISO 9999:2022), National standards for assistive product in China (GBT16432-2016), APs database (18), EASTIN (19) ATAust (20) etc. In addition, rich study also reflect the positive attitude of the academia toward assistive products research. According to the six domains proposed by APL (16)., mobility aids (21–29) have received the most attention in current research, followed by visual (30–33), hearing (34), cognition (35), and environment (36–39) APs. The above confirms that stakeholders have made appreciable efforts in the accessibility of APs, but what is not optimistic is that the high abandonment rate of Aps remains the recent consensus (3–5). Reasons for older adults to abandon the use of assistive devices often include personal factors [e.g., health status (4), Ethics (40), Privacy (41) stigma (42), unmet needs (43)], intervention factors [e.g., design (44), function (45) and services (44)] and environmental factors [e.g., social (46) and discriminated (4)].

It is interesting to note that these findings are coincidentally related to the psychological needs or user experience (47) of the older adults. Preference as a user experience element can improve user acceptance of a product (48). In other words, identifying user preference factors can reduce product abandonment rates. Numerous studies indicated that preferences were key factors for New Product Development (NPD) (49–53). However, the needs and preferences of the older adults are very different from other age groups (54). High-speed aging also puts new demands on the development of APs (55). Although preferences are important, little is known to date about preferences for assistive devices for older adults (56–59). In particular, to our knowledge, there is no study that scientifically captures older adults' preference factors and effectively translates them into assistive device design.

In addition, recent research on design of Aps (APD) suggest that producers should weaken the targeting of their products in order to preserve the dignity of the users and make the products actively used (6, 60, 61). This is right in line with the principles of universal design (similar terms arising from different social cultures includeBarrier-free design, inclusive design (62), design for all These approaches all take the needs of a broader spectrum of people into account in the design process (63). Extensive research has proven that universal design (UD) facilitates social participation (54, 64, 65) and the implementation of active aging policies (54) but the challenge is that UD is difficult to implement in the enterprise (65).

Therefore, it is necessary to establish a specific framework in APD that can facilitate the implementation of UD. To date, previous studies have attempted to establish a number of APD methods aimed at increasing the use of APs. A part of scholars applied the existing single method, technique or principle to the development and design of APs and implemented cases for validation. For example, participatory design (66), synesthetic design (67), Quality Function Deployment (QFD) (68), AHP (69), Sensory Substitution (SS) (70) and Makerspaces (71). Some other scholars have attempted to integrate different well-established methods to create a completer and more integrated framework. Hwang and Park (72) proposed the knowledge of DHSfXs to create alternative solution concepts for assistive device design teams based on 77 Design Heuristics (73). Xassess Teresa's team has built Xassess, an evaluation tool for assistive product design from an interdisciplinary perspective (74). Santos and Silveira (75) integrated user-centered design and additive manufacturing technologies to establish the APD method called AT-d8sign (75). This provides a low-cost and DIY framework for assistive technology design (50). Other studies on the APD framework include a Fuzzy Kano-AHP-DEMATEL-QFD Approach (76), QFD-ANP (77), Usability Context Analysis (UCA) SWOT Analysis TOWS matrix (37), Axiomatic Design (AD) and Theory of Solving Inventive Problems (TRIZ) (78). In the two APD research paradigms above, the use of independent methods is more in-depth and specific, but cannot cover a more complete design process. Whereas, an integrated framework may enable the independent methods to complement each other's strengths, most of the research on integrated frameworks has not been validated by effective cases. In addition, these APD studies barely include the preference factors of older adults.

Based on these issues, There are two primary aims of this study: (1) to determine the way to analyze the preference factors of older adults for APs. (2) to develop a new APD integrated design framework and validate its effectiveness using case studies. This work has dual implications, first it provides a qualitative and quantitative description of older adults' preferences for APs, and meeting older adults' preferences in the early stages of design can be a good way to reduce APs abandonment rates. Incorporating UD principles into research can expand the diversity of user groups and thus expand the market for APs. This bolsters the notion expressed in the WHO report that consumer electronics and assistive technology are integrating more and more (79). Second, an integrated APD framework is more robust and conducive to the practice of assistive product design. This promotes the social participation and active aging of older adults.

## 2. Theoretical background

### 2.1. Preference-based design and Miryoku engineering

Preferences refer to an individual's attitude toward a set of objects, typically reflected in an explicit decision-making process (80). In the consumer decision process, the user's preference factor is Attractiveness of the products. This is a key factor in consumer purchase decisions (81). In order to develop attractive products and systems, Junichiro Sanui and Masao Inui proposed Miryoku Engineering as a preference-based design technique in 1985 (81). It later became a part of Kansei Engineering (82), but Miryoku Engineering places a greater emphasis on customers' subtle inner feelings (53). Evaluation Grid Method (EGM) is the key method used to extract user preference factors in the Miryoku Engineering system (83).

The main purpose of Evaluation Grid Method is to thoroughly explore users' inner feelings to extract details of consumers' cognitive structures and to convert them into concrete factors of assessment as a basis of design (53). EGM is a semi-structured interview method developed by Junichiro Sanui (84) based on Kelly's Personal Construct Theory (85). It is implemented in the following steps:

1. The original evaluation items (OEIs) of samples were obtained by asking respondents for pairwise comparisons.
2. Laddering technique was used to extract abstract evaluation items (AEI) and concrete evaluation items (CEI) on the basis of OEI.
3. Visualize the above cognitive structure.

Researchers have validated the effectiveness of the EGM in a variety of fields. Research in the field of product design is the most abundant. Ma et al. (53) used EGM to analyze consumer attractiveness to 3C products and proposed design strategies for new product attractiveness (53). Xi et al. (86) analyzed the form attractiveness of electric vehicle (BEV) (86). Ko et al. (87) analyzed the effect of personality traits on consumption preferences using office chairs as an example (87). Zhang and Li (83) measured consumer attraction factors for green products aimed at promoting environmental protection (83). Wei and Ma (88) evaluated the elements of attractiveness in the design of attractive children's books (88). INOUE studied the needs of multiple users in mechanical pencil design (89). Wu et al. (90) used EGM to establish a design strategy for Healing Products (90). Research on EGM in other areas including space (91–93), digital products (94–96), events (97, 98), behavior (99, 100), experience (101) etc. The above literature is a sufficient proof of the effectiveness of EGM for extracting user preferences. However, so far, research on the use of EGM for assistive devices is still limited.

In this study, EGM was used to extract the preference structure of older adults for APs in the first phase of the APD framework. Thus, the diverse needs of the older adults can be accurately understood in the early stages of design.

### 2.2. Quantification theory type I

Quantification Theory Type I (QTT1), proposed by Hayashi in 1976 (102), is a multiple regression analysis designed to assign values to qualitative data. Nagamachi uses QTT1 in Kansei Engineering to analyze qualitative data such as consumer feelings and images (82). In addition, a large number of studies have used QTT1 to analyze the weights of qualitative data generated by EGM (83, 96, 98, 100, 103). Techniques such as multiple linear regression (104), Taguchi's method (105), and conjoint analysis (106) have also been used to explain the relationship between the independent and dependent variables. but, QTT1 is simpler and more effective. A review of previous studies revealed that when analyzing qualitative data of EGM, the results of QTT1 are a good representation of the weighting relationships between subjective demands, objective attributes and sub-attributes. However, contradictory relationships often exist in these data. For example, some studies have shown that the contradiction increases with the design attributes (54). The older adults also have contradictions when using APs (107). These contradictions, which may influence the next design decision, need to be resolved in a reasonable way. However, this has hardly been discussed in past studies. In the present study, QTT1 was used in the first phase of the APD framework to quantify the preference factors of older adults. The calculated results served as the basis for the design ideation of the second phase. In addition, contradictory relationships in the QTT1 results were further discussed to fill the research gaps.

### 2.3. Theory of innovation problem solving (TRIZ)

TRIZ, a term from the Russian acronym, is a theory for solving inventive problems proposed by the Soviet engineer Grich Altshuller in 1946 (108). TRIZ solves inventive problems by using a structured approach to identify and eliminate contradictions (109).

TRIZ defines two types of contradictions: Physical Contradictions (PCs) (the direct opposition of two parameters formulated by one and the same system) and Technical Contradictions (TCs) (a situation in which the improvement of a parameter A leads to the deterioration of a parameter B) (110). PCs are solved by the separation principle while TCs are solved by the contradiction matrix and 40 invention principles. Afterwards, the TRIZ invention principles are combined with the domain knowledge of the experts to generate innovative solutions that meet customers' requirements.

TRIZ is considered to be one of the most effective tools for conceiving engineering designs and solving problems (111). TRIZ can effectively improve the novelty and diversity of ideation (112). Also, TRIZ is proven to be a good method for solving problems involving contradictions (113).

The value of TRIZ has been proven in a wide range of research area. In the field of product design, TRIZ, which has been applied to the study of medical equipment (114), Sustainable Product (115), service design (116), eco-design (117), Mechanical design (118, 119), conceptual design (120), Cultural and Creative Design (121), Biologically Inspired Design (122), Structural Design (123), Ergonomic analysis (124), Design Education (125), aims to enhance the reliability of the innovation process based on the scientific method.

Although TRIZ is a powerful tool for design conceptualization, it does not seem to be able to establish the key issues of innovation, nor does it provide a method for evaluating alternatives (126). Therefore, in many studies TRIZ is used in combination with other methods, such as QFD (127, 128), Axiomatic Design (129), Text Mining (130), Genetic Algorithm (130) TOPSIS (115), DEMATEL (131) and fuzzy theory (126), as a more complete framework in the innovation process. Few studies have integrated TRIZ with EGM and QTT1 to study preference-based design.

TRIZ theory contains many tools, and the present study uses several of the most widely used and effective TRIZ tools in the field of product design research including the 40 principles of invention, the contradiction matrix, and the separation principle. In order to propose innovation guidelines for APD in the second phase. The implementation process is as follows.

1. Identify specific contradictions in design elements and translate them into TRIZ contradictions (TC and PC).
2. Resolve technical contradictions (TCs): use the contradiction matrix and the invention principle.
3. Resolve physical contradictions (PCs): use the separation principle (including spatial separation, temporal separation, conditional separation and overall local separation) combined with the invention principle.
4. Propose a specific innovation strategy: propose a specific innovation strategy based on the broad invention principle.

### 2.4. Finite structure method

The main framework of this study references design thinking (132), a process of exploration based on divergent-convergent logic (133). The product function is defined after the TRIZ proposed innovation strategy. However, different combinations of the main functions and sub-functions of a product can take various forms (134). Appropriate methods based on divergence-convergence are needed to achieve a more rational product form.

Finite Structure Method (FSM), a method that can change the spatial layout of a product is used in the framework of this study, which can obtain a rational layout of product functions and provide support for product form design. This is the consensus of several studies (134–137). In this paper, FSM is used to generate various layouts of APs functions. The specific operation steps of FSM are shown as follows:

1. Identify a finite number of functional modules for the target product.
2. Disperse the possible layouts in 2D or 3D geometries.
3. Converge various layouts based on design goals and feasibility to obtain the best solution.

### 2.5. Morphological charts

Morphological charts are design tools for generating integrated conceptual design solutions for design problems in a systematic and analytical manner (138, 139). Theoretically, at least hundreds of specific concepts can be obtained by using morphological diagrams to disperse the product sub-functions. Since its introduction by Zwicky (140), morphological charts have been used in a variety of research areas, including Sustainable Design (141), Human Factors Design (142), Conceptual Design (143), Product-Service System design (144). In addition, the use of morphological charts in combination with other methods [e.g., QFD (145), ANP (146), TRIZ (147, 148) and Fuzzy evaluation (135)] into a hybrid framework is also a popular research paradigm. However, the combination of methods included in this study is different from existing studies, especially the use of morphological charts for the design of assistive devices for the older adults is limited. The specific procedures for the morphological charts in this work are taken from the Delft Design Guide (139) published by Delft University of Technology.

### 2.6. Analytic hierarchy process

Analytic Hierarchy Process (AHP) (149) is the most popular multi-criteria decision making (MCDM) tool invented by Saaty (150). As a decision analysis technique, it can evaluate complex multi-attribute alternatives between one or more decision makers (151). The literature shows that AHP is mainly used to select the best concept among the generated alternatives in the design field (152). The detailed steps of AHP are shown below:

1. Define the problem and determine its objectives, evaluation criteria, evaluation objects and construct their decision models.
2. Each evaluation criterion was scored by pairwise comparisons and each alternative was scored according to each evaluation criterion.
3. Build their comparison matrix.

(1) $$\begin{array}{l} \text{C.R.}=\frac{C.I.}{R.I.},\text{C.I.}=\frac{{\text{λ}}_{\max}-n}{n-1} \end{array}$$

4. Calculations are performed to find the maximum eigenvalue, consistency index C.I., consistency ratio C.R. and normalized value for each criterion/alternative. The algorithm for the consistency relationship is shown in Equation (1). R.I. is the random index. If the maximum eigenvalues (λ_max_), C.I. and C.R. are reasonable (C.R. <0.1), the decision is made based on the normalized values; otherwise, the comparison matrix should be checked for logical errors until these values pass the consistency test.

AHP has been widely used in the design field. such as determine the importance weights for the customer requirements (153), product technical requirements (PTRs) (154), Customer-driven product design process (155), evaluate design concepts (156), select the optimum green product design (157), Analyze product style (158), modular product design (159), product structure design (160), product-service systems conceptual design (161) etc. The integrated AHP makes more realistic and promising decisions than the stand-alone AHP (162). AHP is mostly used in combination with TOPSIS, quality function deployment (QFD), meta-heuristics, SWOT analysis and data envelopment analysis (DEA) to form an integrated framework. Hsiao (158) combined AHP and genetic algorithms to construct a computational product form design model. Zhu et al. (163) combines AHP, QFD and PUGH for medical device design. Karasan et al. (164) discussed the combination of AHP and DEMATEL for customer-oriented product design. Thus, in light of the foregoing analysis, AHP has been sufficiently proven to be effective. In this study, AHP is used to evaluate the alternatives in the third stage of the design model to obtain the optimal solution.

## 3. Proposed preference-based assistive product design model

**Figure 1:**
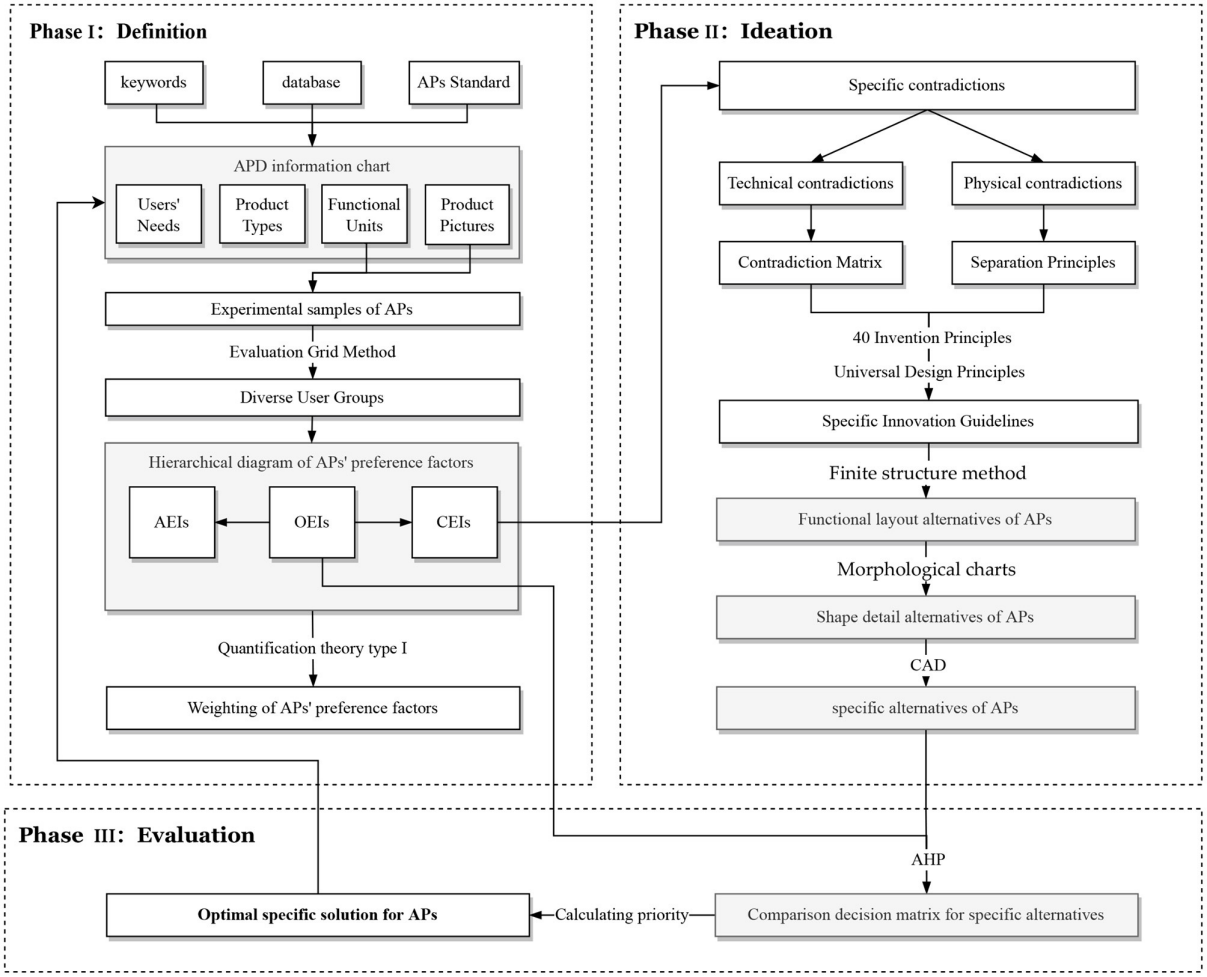
Preference-based assistive product design model (PAPDM).

As described and reviewed in the parts I and II, from a problem-oriented perspective, exploring the factors of older adults' preference for APs is beneficial in addressing the problem of APs abandonment. In terms of methodological orientation, most of the traditional independent methods address a single problem in the design process, and it is also difficult to cover more stages of the design process in the traditional combined form of these methods. In addition, the practicality of UD theory is currently inadequate. For these reasons, this paper proposes a Preference-based Assistive Product Design Model (PAPDM) as shown in Figure 1. The integrated design model consists of three phases: Definition phase, Conception phase and Evaluation phase. The specific implementation steps are shown below:

Phase I: Definition

Step 1: Data collection. Define the design problem, set keywords, select databases (such as integrated search engines, patent databases, research databases, vertical web-sites in the field, etc.) and create an assistive device design information table according to the international and national standards for APs. The APD information charts serves as the basis for subsequent research.

Step 2: Extract the user preference structure. Identify interviewees in diverse potential user groups of the target product and perform semi-structured interviews with them using the experimental samples. Visualize the user preference structure (including OEIs, AEIs and CEIs) using infographics.

Step 3: quantitative analysis. Collect data based on user preference structure by questionnaire method and obtain quantitative data of user preference structure by Quantification Theory Type I (QTT1).

Phase II: Ideation

Step 4: Generate innovation guidelines. The specific contradictions are identified based on the weights in the quantified CEIs, and specific contradictions are trans-formed into TCs and PCs. The innovation guidelines for the target products are generated using contradiction matrix, 40 invention principles, separation principle and UD principles to dissolve the TCs and PCs.

Step 5: Determine the layout of the product functional units. Based on the innovation guidelines, the Finite structure method (FSM) is used to analyze the possible combinations of product functional units and to determine the optimal functional unit layout.

Step 6: The shapes of each functional unit were diverged and converged to obtain several suggested alternatives using Morphological charts based on the functional unit layouts.

Step 7: Identify and visualize alternatives. Use computer-aided design techniques such as 3D or 2D graphic drawing software to visualize alternatives as concrete design alternatives.

Phase III: Evaluation

Step 8: Establish evaluation structure model. Set the OEIs as evaluation criteria and the alternatives as evaluation objects.

Step 9: Calculating weights. Calculate the weight of evaluation criteria and the weight of alternatives under each evaluation criterion.

Step 10: Obtain the optimal solution. Perform final evaluations and obtain optimal solution based on priority.

## 4. Case study

Mobility is a significant consideration in aging and public health research (165). As the population ages, mobility assistive devices are the most common type of APs used by older adults (15, 166), but they are also abandoned more frequently than other categories of Aps (167) for possible reasons such as stigma, inferior quality, and unmet needs as mentioned in section 1 of the paper. Based on the above, we chose walking aids as the target product for the case study, with the aim of demonstrating how PAPDM can be applied in a design scenario. The detailed steps for implementation of PAPDM are listed in the following sections.

### 4.1. Phase I: definition

#### 4.1.1. Data collection

First, the research team set keywords at the beginning of data collection. The terminology of walking aids was referenced to international standards for APs and Chinese standards. The keywords included the near-synonyms (walking frames, rollators, mobility aids), sub-categories of walking aids, and other language descriptions of these words.

Second, appropriate information databases were selected based on the target products, including: comprehensive search engines, patent databases, international competition websites for product design (Reddot, IF, IDEA, etc.), e-commerce websites, scientific research databases, relevant vertical websites, assistive device databases, and self-publishing platforms. Boolean rules were used to collect information using keywords in order to obtain more comprehensive and effective information.

After the data collection, the team members obtained information on 57 walking aids products (including text, images or videos). After removing duplicate, low quality, and low relevance information, 26 typical walker products were obtained, and they were made into a walker product information table. Table 1 shows five of these typical samples. The APD information chart of the walking aid contains the name, picture and functional unit of each product. The research team also analyzed the design strategy of each product, the specific problem solved, and the corresponding TRIZ contradictions and TRIZ invention principles. The APD information chart was the basis for subsequent research.

**Table 1:** The APD information chart of walking aids.

**No**.	**Name**	**Pictures**	**Functional units**	**Design Strategy**	**Specific problems**	**TRIZ contradictions**	**TRIZ invention principles**
1	RAM WALK	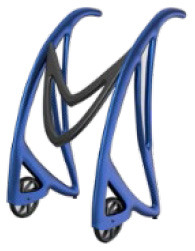	Handles Frame Wheels	Streamlined appearance design	Stigma for the older adults	1, 33	2,13,15,25
2	Indoor Rollator	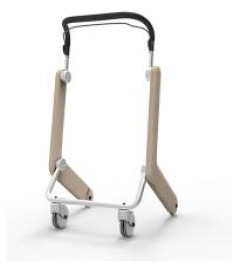	Handles Frame Shaft Wheels	Adjustable handrails	Ergonomic problems	35, 35	Separation Principles
3	TEWL	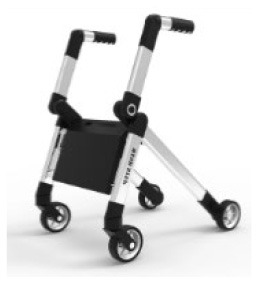	Handles Frame Shaft Seats Wheels	Foldable frame	The inconvenience of storage	8, 22	7
4	Let's Shop	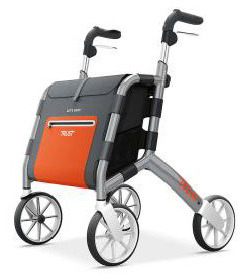	Handles Frame Brakes Baskets Wheels	With soft shopping box	The inconvenience of carrying items when traveling	36, 33	12,17,26,32
5	Tri-Wheel Stair Walker	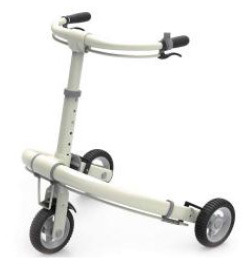	Handles Frame Brakes Wheels	Adjustable wheels	The inconvenience of walking up and down the stairs	35, 35	Separation Principles

#### 4.1.2. Extract the user preference structure

After data collection, the research team identified target users of walking aids as interviewees. It is important to note that judgmental sampling was used instead of random sampling to select the interviewees. This method has the advantage of better cooperation and higher data retrieval rates, as well as greater representativeness. However, it is also important to acknowledge that there are certain limitations to judgmental sampling, as it may introduce bias in the selection of interviewees. In this study, the research team selected interviewees based on both the Universal Design (UD) theory and the Involvement Theory (168). The UD theory was used to consider diverse user groups with direct and potential needs for walking aids to achieve the universal design goals. Additionally, the Involvement Theory was used to select 15 highly involved individuals with relevant experience and expertise, such as their experience in using walking aids, caring for older adults who use walking aids, or their involvement in the design and development of assistive devices. The 15 interviewees (7 males and 8 females) included 4 older adults with more than 2 years of experience in using walking aids, 3 older adults with no experience but weak mobility, 2 older adults who had abandoned their walking aids, 2 older carers, 2 teachers in product design, and 2 experts in assistive device product companies. These interviewees were chosen based on their high involvement and expertise in the subject matter, which aligns with the principles of both the UD and Involvement theories.

Each walking aid product in the APD information chart was made into an A4 size color card. The research team was then divided into groups of 2 to interview the interviewees with 1 person asking questions and 1 person taking notes. First, interviewees were invited to select the preferred card after pairwise comparison of 42 typical walking aid cards and to give the reason (original evaluation items, OEIs) for that sample. Second, the researcher asked interviewees about the abstract reason behind this reason (AEIs) and what concrete reasons were needed to satisfy this (CEIs). This reveals the preferences of the interviewees and the product attributes mapped from these preferences. After the interviews, the researcher compiled the interview transcripts and then a hierarchical preference structure diagram (including 4 AEIs, 7 OEIs, and 26 CEIs) was created. The four colors in this chart represent the three-dimensional structure of the different preference factors in each AEIs. In addition, 7 specific contradictions were extracted from the CEIs after discussion between the research team and the EGM interviewees.

#### 4.1.3. Quantification theory type I

Quantification theory type I was used to calculate the importance between user preference factors, aiming to provide guidance for the second stage of conceptualization. The research team designed the questionnaire based on a hierarchical preference structure diagram. The questionnaire consisted of two main parts; the first part was the basic user information. The second part consisted of four groups of questions (determined by the number of AEIs). Each set of questions includes the importance of each OEI in that AEI and what is the most important CEI. The research team distributed an online questionnaire based on the diverse user groups of EGM interviewees and obtained 105 valid questionnaires. For the calculation of QTT1, OEIs were set as the dependent variable y while CEIs were set as the independent variable x. The quantitative relationship between the two was established by multiple regression analysis. Table 2 shows the results of QTT1. The coefficient of determination R^2^ indicates the reliability of the results, the partial correlation coefficient indicates the importance of OEI, and the category score describes the contribution of CEIs. Positive values in the category scores suggest specific design features that contribute in the associated OEI, while negative values correspond to design features that should be avoided in the ideation phase.

**Table 2:** The results of quantification theory type I.

**OEIs**	**Category (CEIs)**	**Category score (Y1)**	**Category score (Y2)**	**Category score (Y3)**	**Category score (Y4)**
X1	Pcc^*^			0.69405872	
		Z1			−0.694295449	
		Z2			−0.406247362	
		Z3			0.223237356	
X2	Pcc^*^		0.646210977	0.655606618	0.343514246
	Z4		−0.530776453	−0.105372975	−0.258472571
	Z5		−0.199680024	−0.328837861	−0.217732604
	Z6		0.246621294	0.454025079	0.038409297
	Z7		0.059145411	0.573367551	0.038476949
X3	Pcc^*^	0.411489841		0.445619759	
	Z8	−0.429115124		−0.166797472	
	Z9	−0.575073648		−0.321526021	
	Z10	−0.06400715		0.1059421	
	Z11	0.114413793		−0.23622149	
X4	Pcc^*^		0.783922799	0.844469589	0.751018827
		Z12		−0.22390375	−0.978848586	−0.308457724
		Z13		0.126382302	−0.354969171	−0.606445914
		Z14		−0.204260161	0.054010967	−0.412494032
		Z15		−0.710178192	−0.797802612	−0.457824878
		Z16		0.130849266	0.398199191	0.201549546
		Z17		−0.593295253	−0.031180255	−0.046436436
		Z18		1.467959923	1.952759209	−0.728790554
X5	Pcc^*^	0.529768572	0.83239666		
		Z11	0.380356751	−0.391423165		
		Z18	0.893470153	0.04029979		
		Z19	−0.345391974	−0.44752543		
		Z20	0.851427817	0.078373061		
		Z21	−0.179303222	1.224694654		
		Z25	−0.308202661	0.04029979		
X6	Pcc^*^	0.565455518			
		Z18	0.108979596			
		Z21	0.095287889			
		Z22	−0.628173088			
X7	Pcc^*^	0.467701856	0.65057751	0.665737262	0.674195586
	Z23	0.140021593	−0.030294467	−0.003485933	−0.454739207
	Z24	−0.675914602	−0.28965804	−0.574766544	−0.00462502
	Z25	−0.468641009	−0.121455953	0.192825793	−0.047351233
	Z26	0.032813053	0.761661107	0.492385408	0.470048427
Constant term		14.8	14.8	16.8	11.8
R		0.736	0.910	0.891	0.853
Coefficient of determination (R^2^)		0.542	0.828	0.793	0.727

^*^represents the partial correlation coefficient.

### 4.2. Phase II: ideation

#### 4.2.1. Generate innovation guidelines

**Figure 2:**
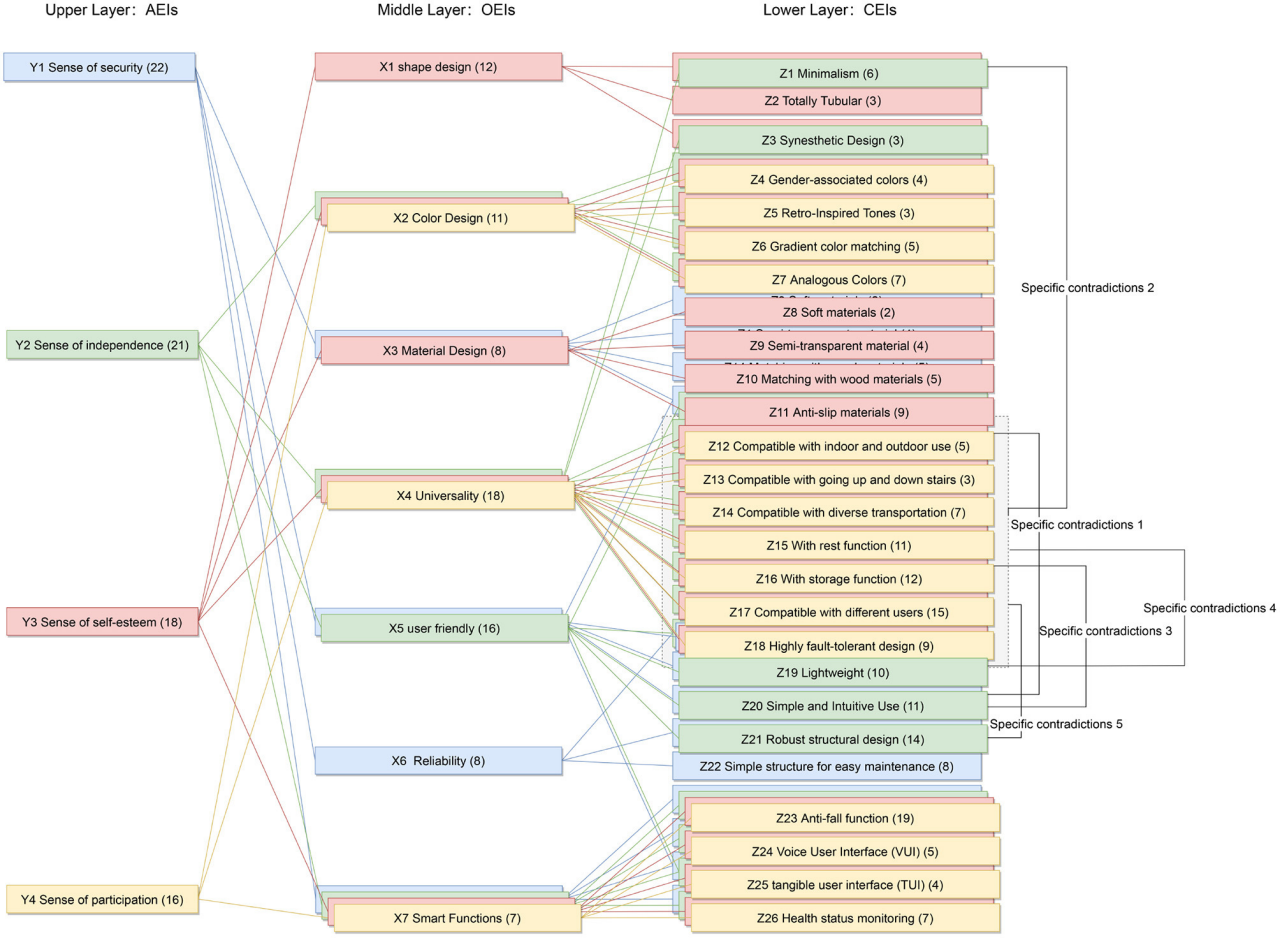
3D Hierarchical structure of preference factors for walking aids.

At the beginning of the ideation phase, TRIZ was used to generate innovation guidelines. First, the research team analyzed seven specific contradictions in the EGM results (Figure 2) based on the results of QTT1 (Table 2). Five specific contradictions were finally identified and transformed into TRIZ contradictions. These 5 contradictions included 3 technical contradictions (TCs) and 2 physical contradictions (PCs). Next, the TRIZ invention principles of these contradictions were obtained based on the contradiction matrix and the separation principle (169). Finally, the research team used these TRIZ invention principles, UD principles, specific design strategies from the APD information chart and their own design experience to identify five specific innovation guidelines (including “Integration of different sizes of wheels”, “Modular design”, “Height- adjustable storage box”, “Partial replacement of accessories” and “Restrictive structural design”). Table 3 shows the detailed process of how the specific contradictions were translated into innovation guidelines.

**Table 3:** Innovative guidelines generation process for walking aids.

**Specific contradictions of walking aids**	**Type of contradiction**	**Engineering parameters**	**Separation principles**	**TRIZ invention principles**	**Innovation guidelines**
Z12	TC	35	N/A	1, 15, 16, 34	Integration of different sizes of wheels
Z20		33			
Z1	PC	36	Conditional Separation	15, 34, 10, 9, 11	Modular design
Z14					
Z16	TC	10	N/A	36, 38	Height-adjustable storage box
Z20		22			
Z15	TC	1	N/A	3, 8, 15, 29	Partial replacement of accessories
Z19		35			
Z17	PC	13	Time Separation	15, 34, 10, 9, 11	Restrictive structural design
Z21					

#### 4.2.2. Determine the layout of the product functional units

**Figure 3:**
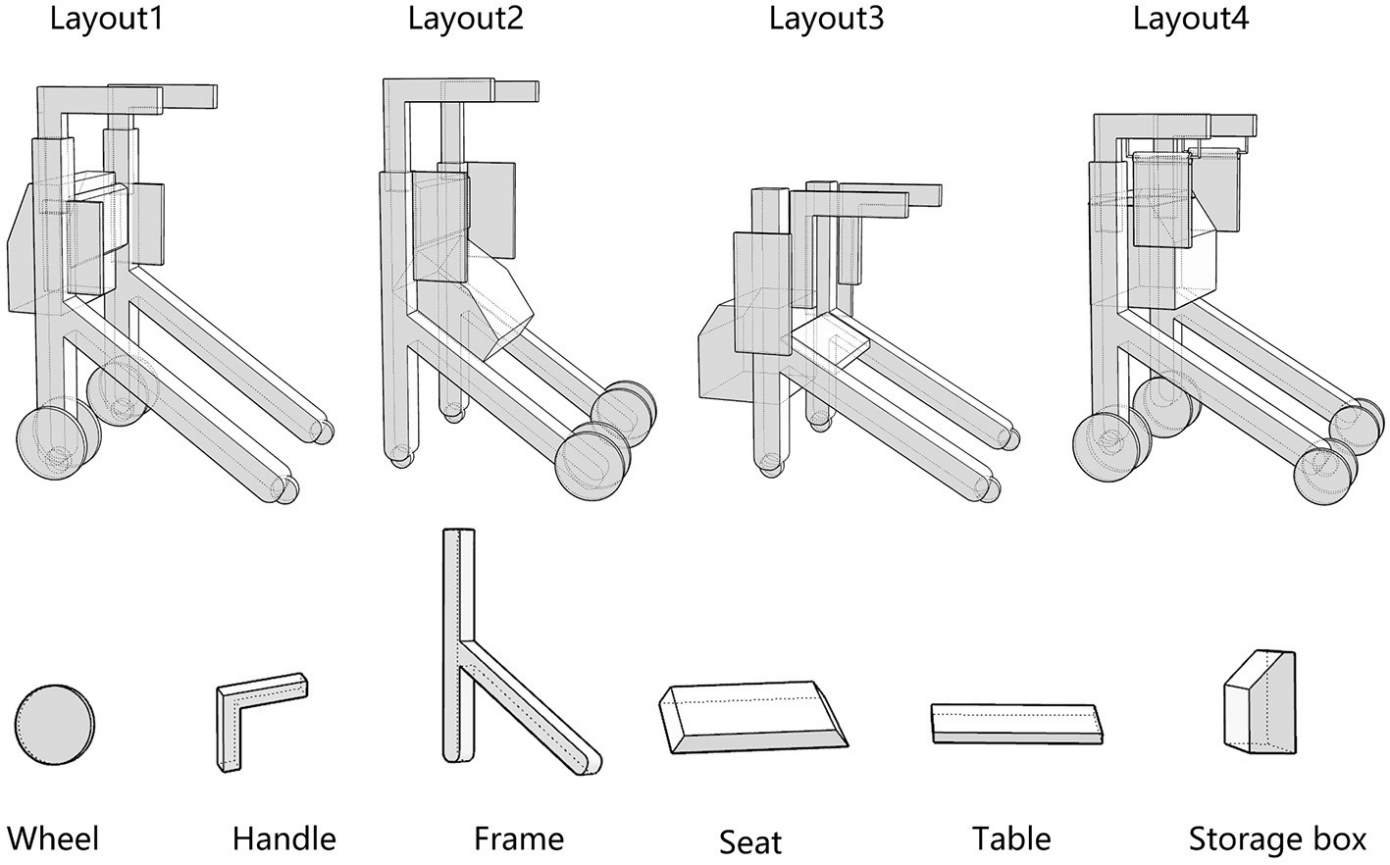
Several functional unit 3D layouts of walking aids.

Innovative guidelines for walking aids are to be achieved through specific functional units and their different layouts in the product. FSM was used to analyze and determine the layout of each functional unit of the walking aid, which also provides the basis for the appearance of the walking aid. First, this study identified six functional units of walking aids including wheels, handles, frames, seats, tables, and storage boxes based on the functional units of each walker in the APD information chart and similar functional units commonly used in other types of products. Next, different shapes were used to represent the corresponding units. The arrangement and combination of the size, number and different placement of the functional unit can provide sufficient form divergence. Finally, the research team extracted four layouts. Finally, the research team extracted four 3D layout options after discussion, as shown in Figure 3. In Layout 1, the front wheels of the walking aid have two kinds of rollers of different sizes, which are convenient for switching between indoor and outdoor. The table is connected to the frame for easy pull-out use, while the seat can be folded for easy storage, but it is more complicated to use. Layout 2 changes the angle of the storage box to facilitate access to items. The rear wheels are large wheels, increasing the stability of outdoor movement, but may affect the indoor use. Layout 3's functional units were arranged more compactly to help save space. Small rollers facilitate indoor use but reduce the stability of outdoor use. The table board is located on the side of the frame to facilitate folding. In Layout 4, the height of the tabletop is conducive to standing use, but may be disturbed by the seat and storage box.

#### 4.2.3. Analysis of product shape details

In this step, morphological charts were used to explore the possibilities of each functional unit. The research team diverged the six functional units in Figure 3 by referring to the APD information sheet of the walking aid and the design mood boards of other products (e.g., Pinterest, Behance, and other design inspiration sites). Several types of each functional unit were then obtained and constructed into a morphological chart (Table 4). In this chart, the permutations of the different types of functional units can generate up to 5 × 4 × 2 × 5 × 3 × 3 = 2000 design solutions.

**Table 4:** The morphological chart of walking aids.

**Function units**	**Type 1**	**Type 2**	**Type 3**	**Type 4**	**Type 5**
Wheel (W)	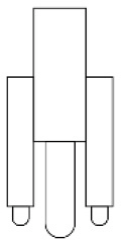	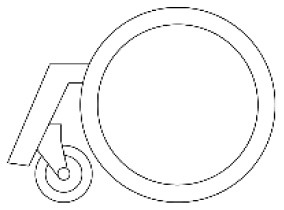	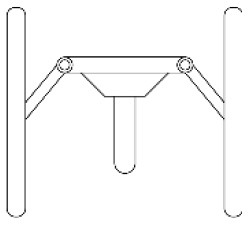	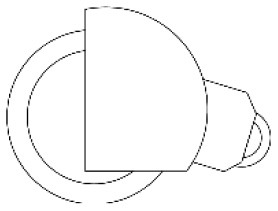	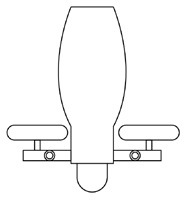
	(W1) Sliding	(W2) Rotating	(W3) Folding	(W4) Semi-wrapped	(W5) Flipped
Handle (H)	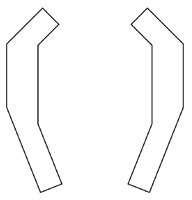	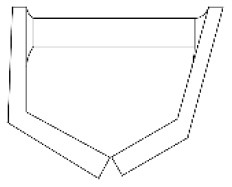	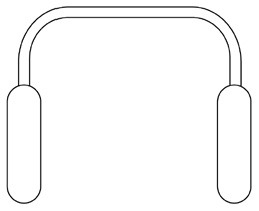	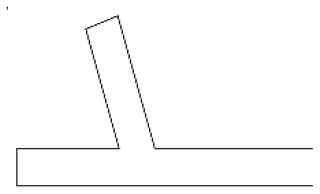	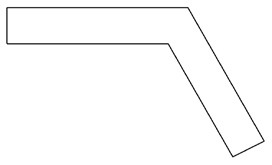
	(H1) Bracket-shaped	(H2) Polygonal	(H3) Chamfered	(H4) Branch-shaped	(H5) obtuse angle
Frame (F)	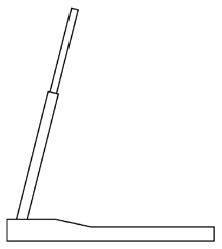	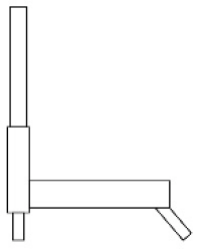	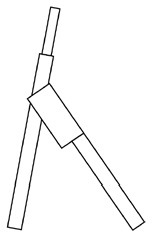	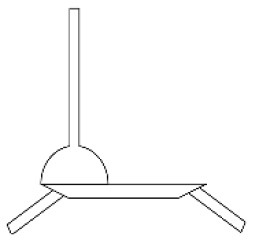	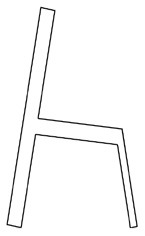
	(F1) Acute angled	(F2) Right-angled	(F3) Branch-shaped	(F4) Trapezoidal	(F5) H-shaped
Seat (S)	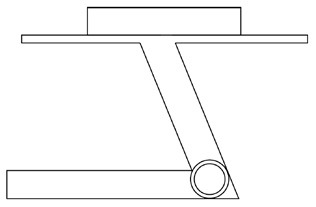	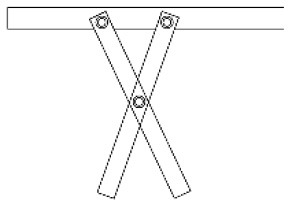	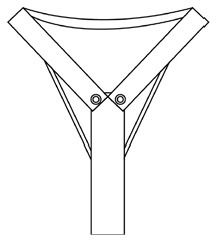	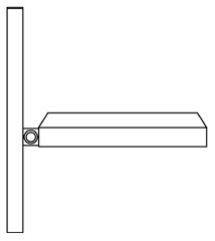	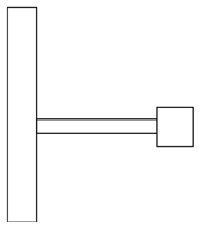
	(S1) Z-shaped	(S2) X-shaped	(S3) Y-shaped	(S4) Folded	(S5) Pull-out
Table (T)	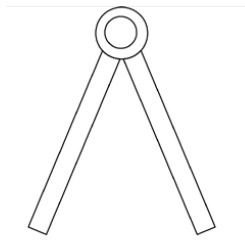	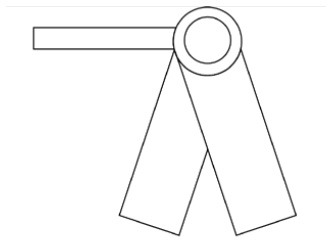	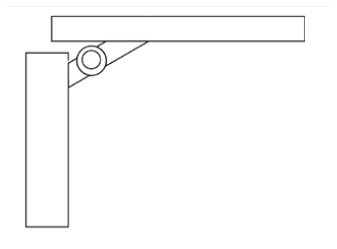	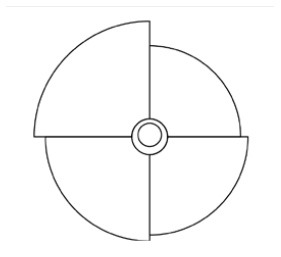	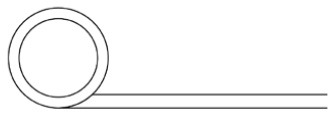
	(T1) Folio	(T2) Revolving	(T3) flipped	(T4) Semi-wrapped	(T5) Rolled up
Storage box (SB)	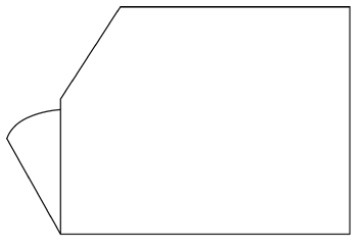	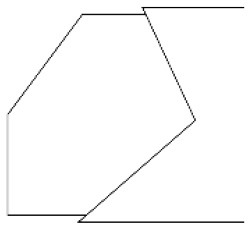	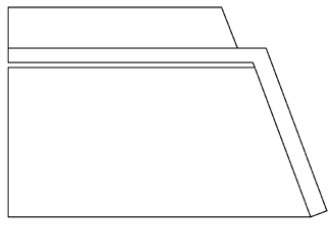	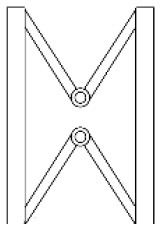	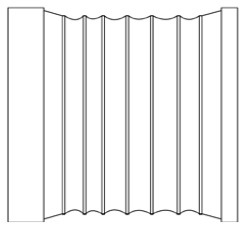
	(S1) Polygonal	(S1) Vertical stack	(S1) Horizontal stacking	(S1) Bi-directional folding	(S1) Bellow

#### 4.2.4. Identify and visualize alternatives

**Figure 4:**
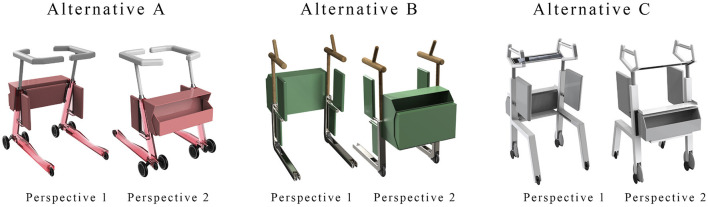
Three alternatives for the walking aids.

The designers of the research team constructed three alternatives (Figure 4) based on the analysis of the advantages and disadvantages of the layout (Figure 3) and morphological details (Table 4) of the walker. Alternative 1 consisted of W3, H1, F1, S4, T3 and SB2. Alternative 2 consists of W4, H4, F2, S4, T3 and S1. Alternative 3 consists of W5, H2, F5, S4, T3 and S3. Finally, the CAD software RHINO 7.0 was used to create the visual 3D model and Keyshot 11 was used to apply color, material and lighting effects to the 3D model.

### 4.3. Phase III: evaluation

#### 4.3.1. Establish evaluation structure model

**Figure 5:**
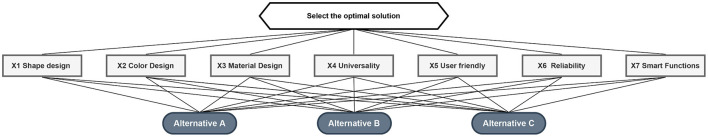
The evaluation structure model of walking aids.

The purpose of the evaluation phase was to select the optimal solution by calculating the priority of the alternatives. According to the PAPDM (Figure 1), the research team used seven OEIs from the 3D Hierarchical structure of preference factors for walking aids (Figure 2) as evaluation criteria to evaluate the three alternatives (Figure 4). The hierarchical structure of the AHP is shown in Figure 5.

#### 4.3.2. Calculating weights

The decision matrix for the seven evaluation criteria was created by the research team through discussion using pairwise comparisons as shown in Table 5. The geometric mean method was used to calculate the priority of the evaluation criteria. The priorities in Table 5 are the normalized results of the geometric mean. It can be seen from the results that criterion X4 is the most important, followed by X6. In appearance design X1 has to be more important than X2 and X3. The lowest weight is given to X3. After the consistency calculation C.R. = 0.043 <0.1 which passed the consistency test.

**Table 5:** Decision matrix for 7 evaluation criteria.

	**X1**	**X2**	**X3**	**X4**	**X5**	**X6**	**X7**	**Priorities**
X1	1	3	5	0.333	2	0.5	2	0.154
X2	0.333	1	3	0.2	0.333	0.5	2	0.075
X3	0.2	0.333	1	0.143	0.2	0.167	0.333	0.030
X4	3	5	7	1	3	2	4	0.340
X5	0.5	3	5	0.333	1	0.5	1	0.115
X6	2	2	6	0.5	2	1	4	0.213
X7	0.5	0.5	3	0.25	1	0.25	1	0.072

Decision matrix after obtaining the decision matrices for the seven evaluation criteria, the research team compared and scored all the alternative solutions in pairs based on each evaluation criterion and subsequently created the decision matrix of alternative solutions as shown in Table 6. The results of the consistency calculation using Equation (1) showed that all alternatives passed the consistency test. This indicates that there are no logical problems with the weights of the 3 alternatives.

**Table 6:** Decision matrix of 3 walking aid alternatives under 7 evaluation criteria.

	**A**	**B**	**C**	**Priorities**	**Criterion**	**C.R**.	**Consistency check**
A	1	2	0.333	0.238	X1	0.016	accept
B	0.5	1	0.25	0.136			
C	3	4	1	0.625			
A	1	0.5	0.5	0.196	X2	0.046	accept
B	2	1	0.5	0.311			
C	2	2	1	0.493			
A	1	0.5	2	0.311	X3	0.046	accept
B	2	1	2	0.493			
C	0.5	0.5	1	0.196			
A	1	0.333	0.5	0.163	X4	0.008	accept
B	3	1	2	0.540			
C	2	0.5	1	0.297			
A	1	0.5	0.333	0.163	X5	0.008	accept
B	2	1	0.5	0.297			
C	3	2	1	0.540			
A	1	2	0.5	0.311	X6	0.046	accept
B	0.5	1	0.5	0.196			
C	2	2	1	0.493			
A	1	0.5	0.5	0.238	X7	0.046	accept
B	2	1	0.5	0.136			
C	2	2	1	0.625			

The final priority values for the 3 alternatives are obtained by the weighted sum of the priorities of the evaluation criteria and the priorities of the alternatives. Table 5 shows the weight matrix β of the evaluation criteria.


$$\begin{array}{l} \beta =\left[0.154\;0.075\;0.030\;0.340\;0.115\;0.213\;0.072\right]\; \end{array}$$

The weight matrix α for the 3 alternatives can be obtained from Table 6.


$$\begin{array}{l} \alpha =\left[\begin{array}{ccccccc} 0.238\; & 0.196\; & 0.311\; & 0.163\; & 0.163\; & 0.311\; & 0.196\\ 0.136\; & 0.311\; & 0.493\; & 0.540\; & 0.297\; & 0.196\; & 0.311\\ 0.625\; & 0.493\; & 0.196\; & 0.297\; & 0.540\; & 0.493\; & 0.493 \end{array}\right]\; \end{array}$$

The priority of the alternative is denoted by S. The result is calculated as follows.


$$\begin{array}{l} S=\alpha \circ \beta =\left[\begin{array}{ccccccc} 0.238\; & 0.196\; & 0.311\; & 0.163\; & 0.163\; & 0.311\; & 0.196\\ 0.136\; & 0.311\; & 0.493\; & 0.540\; & 0.297\; & 0.196\; & 0.311\\ 0.625\; & 0.493\; & 0.196\; & 0.297\; & 0.540\; & 0.493\; & 0.493 \end{array}\right]\;\\ \;{}^{\circ}\left[\begin{array}{c} 0.154\\ 0.075\\ 0.030\\ 0.340\\ 0.115\\ 0.213\\ 0.072 \end{array}\right]=\left[\begin{array}{c} 0.216\\ 0.341\\ 0.443 \end{array}\right] \end{array}$$

**Figure 6:**
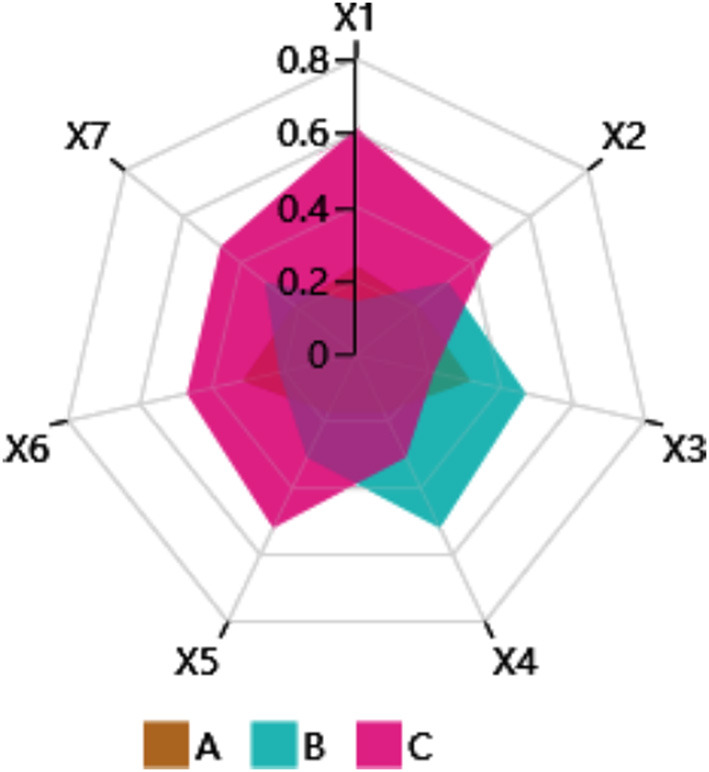
The radar chart of walking aids alternatives.

This calculation shows that the priority of the three alternatives of the walking aid is Alternative C > Alternative B > Alternative A, then Alternative 3 is the best solution. In addition, Figure 6 presents a radar chart of the weight values of the three walking aid alternatives across the seven evaluation criteria in this study. The performance of each alternative on each evaluation criterion dimension is intuitively shown.

## 5. Discussion

The first objective of this study was to identify a suitable method to extract the factors of preference for APs among the older adults as well as other users. The process was based on Miryoku engineering theory, integrating universal design (170) and contradiction analysis (171) from TRIZ based on EGM. Following the traditional EGM process (84), a preference factor structure for multi-user group was created (Figure 2). The advantages of EGM are twofold: first, it not only extracts the objective preferences of users for APs, but also explores the affective preferences behind these specific factors. Compared to similar affective calculation methods such as Kansei engineering (KE) (82) and Kano (172), EGM is easier to operate and effective. Secondly, EGM yields results as a visual cognitive structure that facilitates the enhancement of human thinking (173, 174). It is important to note here that the results of EGM are closely related to participants selection. These results may be influenced by the number of participants, their status, age, experience, etc. Using the judgmental sampling method (175) and involvement theory (168) used in Section 4.1.2 of this paper to select EGM participants is a suggested way.

Compared with traditional EGM methods (83, 87, 99), the improved EGM in this study has several advantages. Firstly, UD theory (170) was considered in the EGM process. The user preference extraction process for Aps involves a wider range of interviewees, including more stakeholders. The aim was to meet the needs of different users through universal design, thereby reducing the abandonment rate of APs and promoting active aging (12). Traditional EGM only interviews target users and pays less attention to the preferences of potential or non-target users, which is contrary to the trend of weakening the targeted development of Aps (6, 60). Secondly, this study added contradiction analysis to the traditional EGM, aiming to provide a basis for innovative ideation. This was also due to the inclusion of universal design. The expansion of the user group leads to greater differences in user preferences and more obvious contradiction between preference factors. In previous related studies (84, 176, 177), the contradiction relationships between lower items (CEI) in EGM were rarely discussed. Chen (95) used the CIP measurement method to divide EGM interviewees into three groups and found cognitive differences among users with different levels of participation. Personality differences (87) and user background differences (178) have also been discussed in previous EGM studies. However, these studies did not mention how to resolve these differences or contradictions in the design process. Thirdly, the research team used 3D charts to visualize the multi-level cognitive structure of interviewees. From Figure 2, it can be seen that the same OEIs and CEIs may be associated with multiple AEIs, so different colors are used to represent the preference factors included in each AEI in the EGM chart. Although some scholars have developed visualization tools for EGM (173, 174), the 3D EGM chart was more intuitive and clear than the 2D EGM chart in previous studies.

The second purpose of this study was to develop an integrated design framework for APD that aligns with the preferences of older adults. The PAPDM (Figure 1) was the result based on this purpose. At the core of this model is divergent and convergent thinking, which is similar to classical design thinking models such as the double diamond model (179). The advantages and possible roles of PAPDM are as follows:

First, PAPDM will help solve the difficulties of information collection in the APD process. This is specifically achieved through the APD information chart constructed during the definition phase. As can be seen in Section 4.1.1, in the first stage, different types of databases were used to collect a wide range of walking aids (Table 1). The images and textual information in the table were the source of representative samples for EGM. Design inspiration was also provided for the implementation of TRIZ, FSM, and Morphological Charts in the second phase. Compared to traditional data collection and sample preparation processes, using this integrated graphic and textual information table helps designers reduce the difficulties of repeatedly searching long-term memory (LTM) and generate more design inspirations. APD information chart (Table 1) produced similar effects to case-based reasoning (CBR) (180) and design heuristics (DHs) (181), as also demonstrated in the research results of Lee et al. (182) and Hwang and Park (72).

Second, PAPDM can clearly elicit the perceptions of user groups such as older adults regarding APs. This is achieved through a hybrid approach of EGM and QTT1. Section 4.1.2 shows the detailed EGM process. On the left side of Figure 2, it can be seen that the emotional preference factors of older adults focus on security, independence, self-esteem, and involvement, which supports the policy recommendations of the Active Aging Framework (12). In the middle layer of Figure 2, X1, X2 and X3 are product appearance attributes while X4-X7 embody the product function attributes. Section 4.1.3 shows the implementation process of the QTT1, and the results in Table 2 show the spatial layout of OEIs and CEIs in different AEIs. The high value of Coefficient of de-termination (R^2^) indicates that the result explains most of the data, which is similar to previous studies (96, 183). It is worth reflecting that the negative values in the scores of CEIs are often not well interpreted and used, which is worth exploring in future studies.

Third, PAPDM makes the conceptualization process of APD transparent. In previous research (83, 96), the results of EGM and QTT1 were rarely further utilized, especially in a progressive and transparent way to generate concepts. In the second stage of PAPDM, TRIZ was used to connect the results of QTT1. The five innovation guidelines proposed in the ideation phase (Table 3) were all functional or structural innovation strategies used to meet multiple needs. This result was also demonstrated in Zhang et al. (126) study. This may be related to the principle of invention chosen by the research team. Each contradictory pair contained multiple invention principles, and the research team obtained a total of 20 invention principles each of which could be specified as multiple innovation guidelines. The final five innovation guidelines were selected on the basis of universal design principles, and the versatility of TRIZ in generating ideas has been demonstrated in a wide range of studies (146, 171, 184). Section 4.2.2 and section 4.2.3 are both about the form divergence of the walking aid. The former analyzes the layout of each functional unit of the walker the latter considers the diversity of design details. In previous FSM studies (185), the layout of functional units was mostly arranged in two dimensions with words, symbols and geometric shapes. 3D layout (Figure 3) facilitates the diversity of solutions. The morphological chart of the walking aid (Table 4) shows that each functional unit was diverged into five unique form details. However, how to converge the ideal solution among the large number of details has not been agreed in previous studies (141, 147) and deserves to be discussed in future studies.

In addition to the advantages mentioned above, an interesting finding is that the AHP methods in the evaluation phase can be well compatible with EGM. They are both based on a hierarchical analysis method. The evaluation criteria in the AHP just correspond to the OEIs in the EGM results. The results of Lu et al. (134) and Kang et al. (186) study also prove this finding. In the evaluation phase, the evaluation criteria were the seven OEIs in the EGM. The priorities of these evaluation criteria (Table 5) are generally consistent with the pcc values demonstrated in the QTT1 results (Table 2). For example, the pcc value of X4 (generalizability) in Table 2 is much larger than the other OEIs. The priority of X4 (0.34) in Table 5 also ranks first. This proves the consistency of the QTT1 and AHP algorithms. In addition, the AHP evaluation criteria can be subdivided into multiple sub-criteria and the evaluation criteria and solution priorities can be used to obtain more accurate results through group decision making. Additional studies are needed to consider these variables.

## 6. Conclusions

This paper was conducted to address two research questions. The first research question addressed how to capture the preference factors of a multi-user group, primarily consisting of older adults, toward assistive products. The research team integrated EGM, UD, contradiction identification, and QTT1 to extract and analyze the preference factors of the user group, including their relationships and weights. The second research question focused on establishing and validating an effective Assistive Product Design (APD) framework based on Universal Design principles. We introduced a preference-based assistive product design framework called PAPDM and demonstrated its detailed process through a case study involving the design of a walking aid. The framework comprised three major stages. The first stage, the definition phase, addressed the first research question. In the second stage, we employed the TRIZ contradiction matrix and the 40 Inventive Principles to propose design guidelines by resolving contradictions within the assistive product design. We explored various solution alternatives using FSM and morphological charts to generate more suitable alternative designs. In the third stage, we utilized the Analytic Hierarchy Process (AHP) to evaluate the alternative designs and make decisions. Both PAPDM and the walking aid solution C from the case study are currently undergoing patent applications for invention and utility models. The case study demonstrated that the APD information chart is an effective way to gather information, compensating for the limited information provided by older adults during interviews.

This project marked the first combination of EGM and TRIZ, and we discovered that these two methods effectively complement each other. They not only enhanced the role of EGM in proposing design strategies but also provided a problem identification approach for TRIZ. TRIZ, in turn, offered innovative guidelines for FSM and morphological charts. Furthermore, PAPDM enhanced the applicability of UD theory in assistive product design to a certain extent.

One limitation of this study was the small number of interviewees, which may have resulted in a limited breadth of coverage in the interview results. User preferences also change with societal development and the iterative process of assistive product design, necessitating regular surveys of larger target user groups. Additionally, the invention principles of TRIZ are abstract and broad, so the elimination process of contradictions is influenced by variations in the quantity and quality of designer knowledge. Further research may be needed to impart disciplinary attributes to the invention principles of TRIZ. Lastly, the evaluation criteria for alternatives were single-layered in structure, and in the future, different sub-criteria could be listed to enable a more accurate comparison of alternative details. Despite these limitations, the research findings in this paper provide a logically robust and actionable framework for fostering innovation in assistive products. This study holds practical significance in improving the independence and social participation of older adults.

## Data availability statement

The original contributions presented in the study are included in the article/supplementary material, further inquiries can be directed to the corresponding author.

## Ethics statement

The studies involving human participants were reviewed and approved by the Ethics Committee of Xiamen University of Technology. The patients/participants provided their written informed consent to participate in this study.

## Author contributions

BZ and MM contributed to conception and design of the study. MM provides the core research methodology in the manuscript. BZ organized the database and performed the experiment. BZ and ZW performed the statistical analysis and wrote the draft of the manuscript. ZW visualized the results of the study. All authors contributed to manuscript revision, read, and approved the submitted version.
